# Complete Genome Sequence of the Engineered *Escherichia coli* SHuffle Strains and Their Wild-Type Parents

**DOI:** 10.1128/genomeA.00230-16

**Published:** 2016-03-31

**Authors:** Brian P. Anton, Alexey Fomenkov, Elisabeth A. Raleigh, Mehmet Berkmen

**Affiliations:** New England Biolabs, Ipswich, Massachusetts, USA

## Abstract

SHuffle strains are genetically engineered *Escherichia coli* strains that are capable of oxidizing cysteines within proteins to form disulfide bonds. Here we present the complete genome of both the K-12 and B versions of SHuffle strains along with their parental ancestors. These strains have been of significant use to both the general scientific community and the biotech industry, interested in producing novel disulfide-bonded proteins that were hitherto unable to be expressed in standard *E. coli* expression strains.

## GENOME ANNOUNCEMENT

Wild-type *Escherichia coli* strains are incapable of producing disulfide-bonded proteins due to the presence of two reducing pathways, the glutaredoxin and thioredoxin pathways. The reductases in these pathways recognize disulfide-bonded proteins and reduce them to maintain the proteome in its reduced state.

We constructed two *E. coli* strains capable of promoting the production of correctly disulfide-bonded proteins in the cytoplasm by genetically knocking-out *gor* and *trxB* (1) and expressing *dsbC* in the cytoplasm. These strains, SHuffle B and SHuffle K-12 (2), were derived from two commonly used laboratory strains, ER2566 (B background) and DHB4 (K-12 background), respectively. Since their introduction in 2009, numerous studies have used SHuffle strains to express various disulfide-bonded proteins (3), including the recent demonstration of the expression of full-length antibodies in bacterial cytoplasm for the first time (4). Surprisingly, SHuffle strains can also express certain non-disulfide-bonded proteins better than wild-type protein expression strains, indicating a need for a deeper understanding of the biology of these strains. The elucidation of the genomes of these strains should therefore lead to a deeper understanding of the biology of the SHuffle strains.

The four strains were sequenced using the Pacific Biosciences platform on an RSII instrument using P6 chemistry. For each strain, a 10-kb SMRTbell library was prepared from total DNA using the manufacturer’s instructions, size-selected (4 to 50 kb) using a BluePippin (Sage Science), and sequenced on 2 SMRT cells with 240-min movies. Assembly was performed using RS_HGAP_Assembly followed by manual refinement and re-sequencing using RS_BridgeMapper. HGAP coverage ranged from roughly 100× to 225×.

The genomic sequence confirmed the genetic deletions of *trxB* and *gor*, but also revealed two unexpected differences: a new allele of the AhpC suppressor and chromosomal duplications of cytoplasmic *dsbC*. Unlike previous studies of *trxB* and *gor* suppression by AhpC* in *E. coli* K-12 (5), the naturally selected suppressor of *trxB* and *gor* in *E. coli* B is a triplet codon contraction and not an expansion. The identity of this novel suppressor should result in an expanded understanding of the redox biology. Furthermore, the genetic insertion of the *dsbC* overexpressing plasmid into the lambda attachment site using lambda InCh (6), should result in a single copy insertion. However, the genomic sequences of SHuffle K-12 (C3026) and SHuffle-B (C3029) revealed a triplicate and a duplicate copy of the insertion, respectively. These illegitimate duplication events could be due to the role of cytoplasmic DsbC in alleviating oxidative stress within the SHuffle strains.

These strains have been of significant use to both the general scientific community and the biotech industry in the production of novel disulfide-bonded proteins that are otherwise inexpressible in standard *E. coli* laboratory strains.

### Nucleotide sequence accession numbers.

The annotated complete genome sequences are deposited at EMBL, EBI, under the following assembly accession numbers: NEB T7 express (C2566), CP014268; SHuffle-B (C3029), CP014269; DHB4, CP014270, and its cognate F′ episome, CP014271; and SHuffle K-12 (C3026), CP014272, and its cognate F′ episome, CP014273.
